# First person – Sachin Wani

**DOI:** 10.1242/dmm.049540

**Published:** 2022-04-27

## Abstract

First Person is a series of interviews with the first authors of a selection of papers published in Disease Models & Mechanisms, helping early-career researchers promote themselves alongside their papers. Sachin Wani is first author on ‘The Paget's disease of bone risk gene PML is a negative regulator of osteoclast differentiation and bone resorption’, published in DMM. Sachin is a research assistant (postdoctoral) in the lab of Prof. Omar Albagha at University of Edinburgh, Edinburgh, UK, where he applies knowledge of biomedical science to the investigation of health, development, biological function and disease.


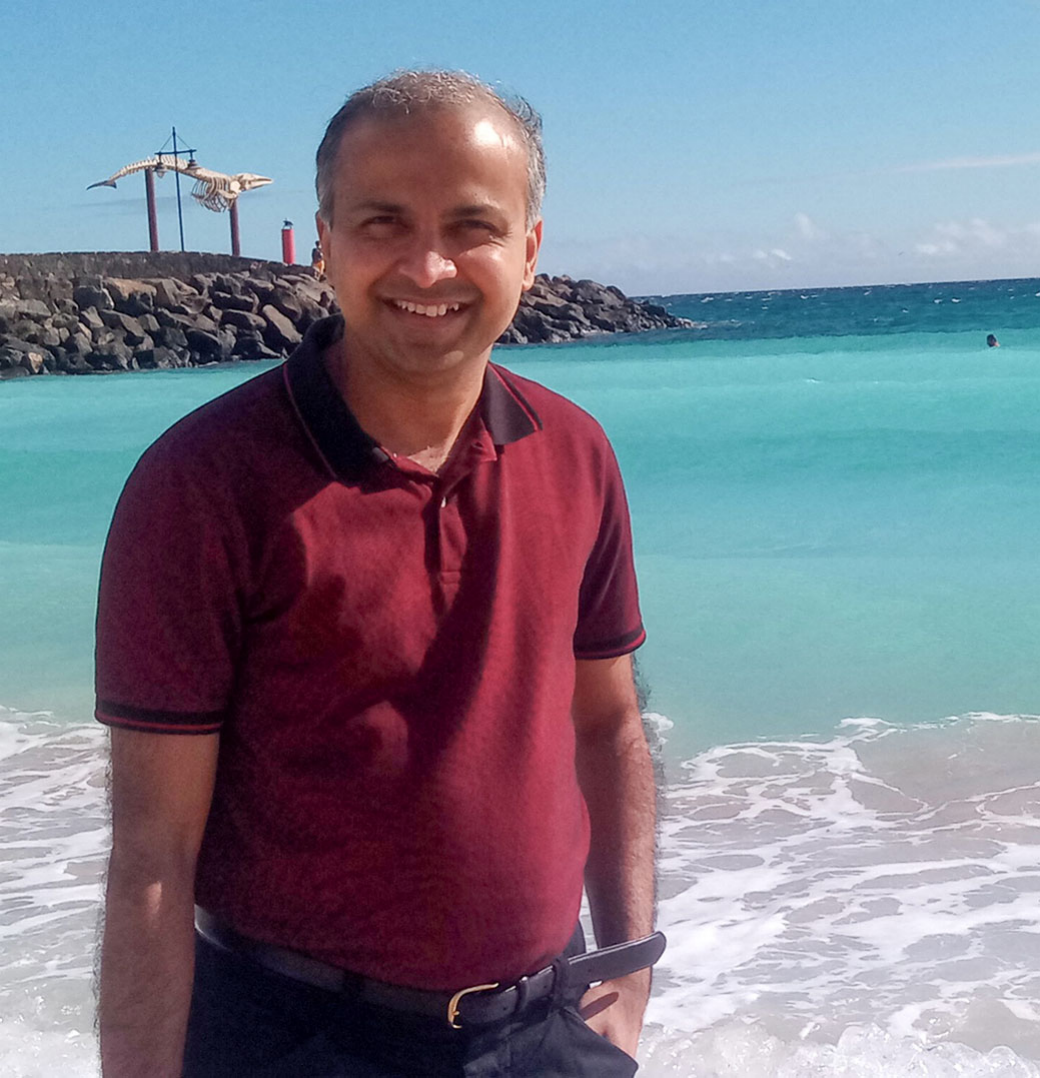


**Sachin Wani**

**How would you explain the main findings of your paper to non-scientific family and friends?**

Bone is a dynamic tissue that constantly undergoes change. The process of bone loss performed by bone cells called osteoclasts is tightly coupled with bone formation by osteoblasts, but in some diseases, such as Paget's disease of bone (PDB), the process is accelerated and becomes abnormal, resulting in abnormal bone architecture. PDB is a common bone disorder affecting specific sites within the skeleton that disrupt bone structure and function, leading to the development of various complications, such as bone pain, bone deformity, secondary osteoarthritis, nerve compression and fractures. It is a disease of old age and exhibits slow progression, often being diagnosed when irreversible skeletal damage has occurred and treatment provides little clinical improvement to the patient. The cause of PDB is not completely understood, but growing evidence suggests that genetic factors play a major role; however, environmental factors, such as viral infection, diet and mechanical stress on the skeleton, have also been reported. We have found that a genetic variant (change in DNA) in PDB patients is associated with reduced expression of the *PML* gene, and blood cells of patients with PDB have decreased amounts of PML compared with healthy individuals. Mice with PML deficiency show increased activity of osteoclasts and osteoblasts. Our study shows that reduction in *PML* expression increases the risk of getting PDB and therefore identifies PML as a novel regulator of bone metabolism.

“Our study [...] identifies PML as a novel regulator of bone metabolism.”

**What are the potential implications of these results for your field of research?**

It improves our understanding of role of PML in bone metabolism, which until now has been known to be mainly involved in tumour suppression. Genetic factors identified at the PML locus could be used in conjunction with other genetic markers to assess individuals with family history, as well as a higher risk of developing PDB so that treatment or disease-modifying strategies can be implemented early in life before permanent damage occurs. It will also lead to a better understanding of PDB pathophysiology, as well as to identifying new molecular and bone signalling pathways, and potential therapeutic targets. It will also help in better understanding and applying this knowledge to disorders with abnormal bone turnover.

**What are the main advantages and drawbacks of the model system you have used as it relates to the disease you are investigating?**

The advantages of our mouse model are the ease of extraction of relevant tissue (bone) and the ability to conduct experiments without restrictions on sample material, unlike research using human material or tissue where bone tissue is very difficult to procure and analyse, especially from healthy individuals; and good extrapolation to human disease. Disadvantages include the physiological state in a human being not being accurately represented in the model, as it is rarely the case that a gene is completely ablated. Murine biology still varies a lot from human.

**Figure:**
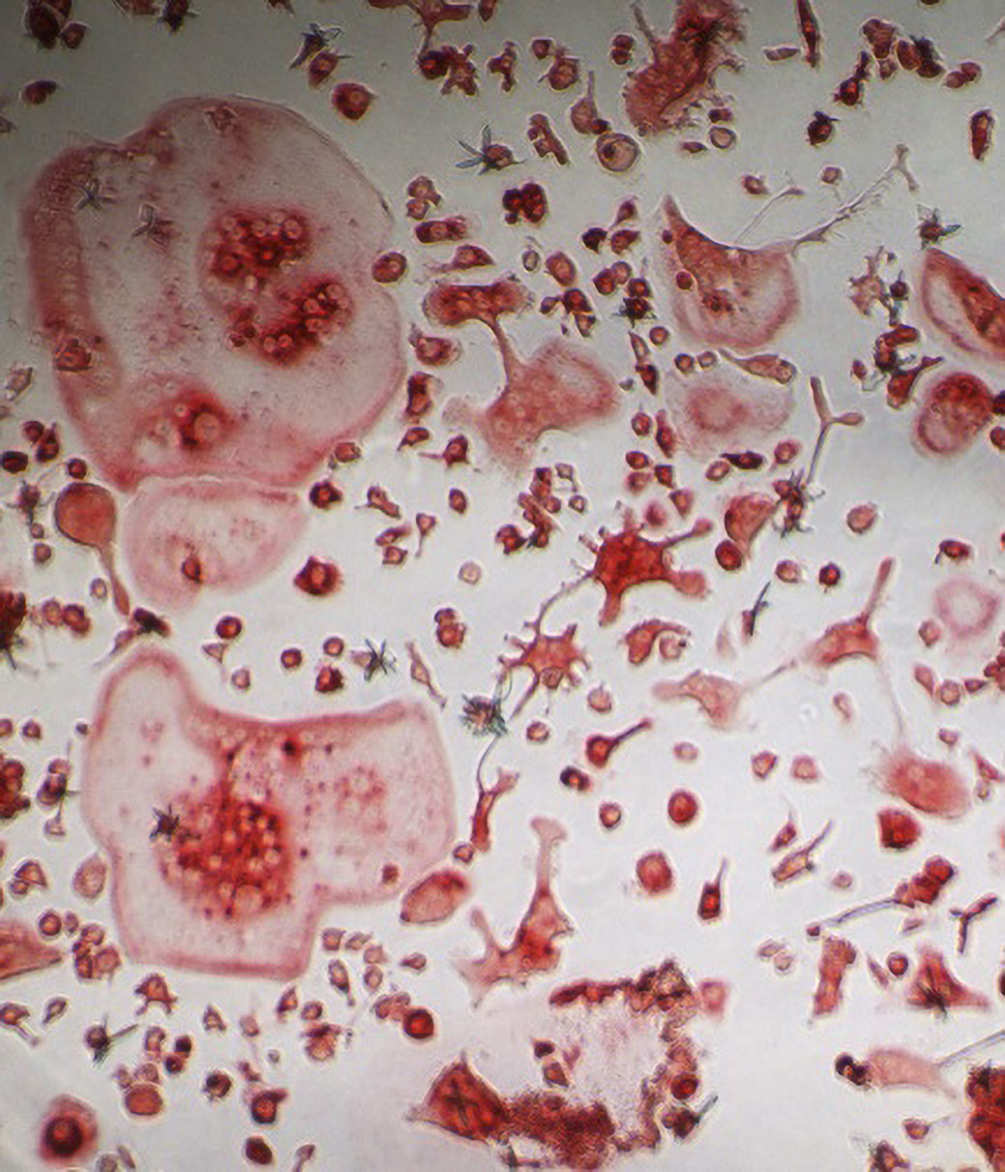
Large and numerous multinucleated osteoclasts (bone-resorbing cells) in mice with PML deficiency.

**What has surprised you the most while conducting your research?**

The main thing is how complex our biological systems are (e.g. so may genes exhibit pleiotropy) and how difficult it is and/or will be to disentangle interaction of genetic and/or biological factors with environmental factors to develop deep understanding of human evolution, health and disease.

**Describe what you think is the most significant challenge impacting your research at this time and how will this be addressed over the next 10 years?**

Research in bone or calcified tissue is not considered lucrative compared with cancer, neurology or cardiac fields, or, for that matter, even infectious disease research now (with emergence of Covid). This leads to lack of sufficient funding in the long term. However, there is still a significant level of morbidity and quality of life issues with many of the bone diseases such as osteoporosis and osteoarthritis, with no obvious cure, so I hope that their importance is not ignored and the governing bodies and research councils continue to fund and promote R&D in the bone disorder field.

**What changes do you think could improve the professional lives of early-career scientists?**

There should be dedicated sources of funding and career paths in university (academia) and private (industry) collaborations to keep the motivation and morale of scientists and allied health staff high. In addition, opportunities for teaching and supervision, as well as regular training and networking events, are needed to keep up to date with the latest in science and technology development. The Covid pandemic has clearly shown us the importance of scientists and what scientific developments can achieve, e.g. Covid vaccines.

**What's next for you?**

I am continuing research in PDB to explore the role of epigenetics and environmental factors in this disease. I am also supervising PhD students now and would like to become actively involved in teaching at multiple levels (undergraduate/postgraduate) using different methods (in person and online or distance learning). I wish to use my knowledge and skills to train and encourage my students to lead a career in science, thereby ensuring that passion for science does not die out. The overall objective is to help bring up a new generation of scientists, and creative individuals and research entrepreneurs who will make the world a better place to live than it already is.
